# Weed detection in soybean fields using improved YOLOv7 and evaluating herbicide reduction efficacy

**DOI:** 10.3389/fpls.2023.1284338

**Published:** 2024-01-11

**Authors:** Jinyang Li, Wei Zhang, Hong Zhou, Chuntao Yu, Qingda Li

**Affiliations:** ^1^ College of Engineering, Heilongjiang Bayi Agricultural University, Daqing, China; ^2^ Key Laboratory of Soybean Mechanization Production, Ministry of Agriculture and Rural Affairs, Daqing, China

**Keywords:** weed detection, YOLOv7, soybean, UAV, herbicide reduction, weeding

## Abstract

With the increasing environmental awareness and the demand for sustainable agriculture, herbicide reduction has become an important goal. Accurate and efficient weed detection in soybean fields is the key to test the effectiveness of herbicide application, but current technologies and methods still have some problems in terms of accuracy and efficiency, such as relying on manual detection and poor adaptability to some complex environments. Therefore, in this study, weeding experiments in soybean fields with reduced herbicide application, including four levels, were carried out, and an unmanned aerial vehicle (UAV) was utilized to obtain field images. We proposed a weed detection model—YOLOv7-FWeed—based on improved YOLOv7, adopted F-ReLU as the activation function of the convolution module, and added the MaxPool multihead self-attention (M-MHSA) module to enhance the recognition accuracy of weeds. We continuously monitored changes in soybean leaf area and dry matter weight after herbicide reduction as a reflection of soybean growth at optimal herbicide application levels. The results showed that the herbicide application level of electrostatic spraying + 10% reduction could be used for weeding in soybean fields, and YOLOv7-FWeed was higher than YOLOv7 and YOLOv7-enhanced in all the evaluation indexes. The precision of the model was 0.9496, the recall was 0.9125, the *F*1 was 0.9307, and the *mAP* was 0.9662. The results of continuous monitoring of soybean leaf area and dry matter weight showed that herbicide reduction could effectively control weed growth and would not hinder soybean growth. This study can provide a more accurate, efficient, and intelligent solution for weed detection in soybean fields, thus promoting herbicide reduction and providing guidance for exploring efficient herbicide application techniques.

## 1 Introduction

Soybean is an important source of high-quality protein and an important grain, oil, and feed crop. With the improved living standards of Chinese residents, the demand for soybeans is increasing. Enhancing soybean production and quality is of great significance to safeguarding the security of the soybean industry and national grain and oil security. Over the past few years, our soybean production has increased significantly. However, weeds continue to be a major problem that hinders soybean yield improvement. Weeds compete with crops for nutrients and water and provide a host environment for pests and diseases, and weed seeds can accumulate in the soil and affect the next year’s crops (Ahmad et al., 2020). Weeding is a key task in agricultural production, which not only improves crop yield and quality but also reduces the use of herbicides and environmental pollution (Teimouri et al., 2022). Chemical weeding has gradually become an important weeding method due to its easy operation and significant effect, but it also brings environmental and food safety problems. Therefore, finding an environmentally friendly and efficient weeding method is an important task in current agricultural production.

In order to solve the problems caused by traditional chemical weeding, herbicide reduction has become a trend. Efficiency reduction and green weeding have been advocated, and herbicide reduction measures have been proposed by researchers (Fang et al., 2022). Herbicide reduction achieves the best weeding effect by using the smallest possible dose of drugs, which improves weeding efficiency, reduces production costs, and minimizes environmental pollution (Wang et al., 2023).

In the process of herbicide spraying, the use of appropriate spraying techniques will dramatically increase the weeding effectiveness. Electrostatic spraying technology can improve the herbicide adhesion rate and control effect and also improve the weeding efficiency and quality. Electrostatic spraying utilizes charging methods such as corona, induction, and contact to make droplets electrostatically charged, which are adsorbed onto crop leaves through directional motion (Lan et al., 2018). In electrostatic spraying, large droplets in the electric field force and surface tension under joint action are broken into smaller droplets, increasing the uniformity of the droplets (Gong et al., 2022). Ru et al. (2015) found that electrostatic spraying increased droplet coverage and liquid deposition in the canopy, middle, and lower layers of the target. Yu et al. (2005) realized that the droplet spectrum of electrostatic spraying was narrower and the effective deposition of pesticides increased significantly. Soybeans can be better protected from herbicide damage through reduced herbicide use and improved herbicide attachment rates. Weeding in soybean fields can be better achieved through a combination of reduced herbicide use and electrostatic spraying techniques.

The traditional assessment of the weeding effect is mainly through manual field survey, and human subjective factors have a large impact and need to enter the field several times, which significantly increases the labor force and low efficiency. UAVs have been tested as a high-throughput crop growth assessment tool with high-resolution, timely and rapid, and wide-area characteristics, providing the means to support on-farm information detection (Feng et al., 2020). UAVs are now improving weed monitoring for different types of crops in a more efficient and environmentally friendly way (Mohidem et al., 2021). In addition, with the rapid development of artificial intelligence technology in the agriculture field, smart agriculture is gradually replacing traditional agricultural techniques that can increase crop yields but are harmful to the environment, such as the excessive use of pesticides and fertilizers. Smart agriculture has become a new form to realize sustainable agriculture development, which can improve the efficiency of agricultural production, reduce production costs, and improve the quality of agricultural products (Tang et al., 2023). Deep learning techniques are playing an increasingly important role in the context of smart agriculture. By training and learning from a large amount of agricultural image and video data, there are many applications in crop image classification, detection, and segmentation, which can lead to a better understanding of crop growth and timely measures for intervention (Wu et al., 2023). Currently, deep learning has been combined with UAV remote sensing technology to automatically extract deep information from UAV-captured images and model complex problems, which has significant advantages in crop identification research in field environments (Rakhmatulin et al., 2021; Rai et al., 2023). The YOLO series algorithm, as a typical algorithm for single-stage target detection, obtains the target area, position, and category of the corresponding object through direct regression, which has the advantage of faster detection speed compared with two-stage target detection algorithms (Jiang et al., 2022). The YOLO series algorithms have been widely used in weed detection and have achieved some research results. Wang Q. et al. (2022) constructed a YOLO-CBAM model by incorporating the attention mechanism in YOLOv5, which can be applied to the real-time detection of *Solanum rostratum* Dunal seedlings in the field with a precision of 0.9465 and a recall of 0.9017. However, the study did not explore the effects of environmental changes, lighting conditions, etc. on model performance. Pei et al. (2022) used improved YOLOv4 to detect weeds in corn fields, introduced the Meta-ACON activation function, added the CBAM module, and replaced NMS with Soft-NMS. The mean of average precision (*mAP*) of the improved model reached 86.89%, but the authors did not explore the effects of different light conditions and different crop growth stages on the model performance. Zhang et al. (2022) proposed an EM-YOLOv4-tiny model based on YOLOv4-tiny fusing multiscale detection and attention mechanisms for weed detection in peanut fields. The *mAP* reached 94.54%, but the recognition range in the image was small, limiting the model application in large field experiments. Other scholars such as Yang et al. (2022) detected weeds in alfalfa through YOLOv3. Fatima et al. (2023) developed a lightweight weed detection mechanism to assist laser-weeding robots through YOLOv5. Liu M. et al. (2022) constructed a weed detection model for maize fields based on the MSRCR-YOLOv4-tiny, which provides a feasible real-time weed identification method for precision weed control systems in fields with limited hardware resources.

In recent years, YOLO has gone through several versions of updates and development, including YOLOv5, YOLOv6, YOLOv7, and YOLOv8. They are constantly driving the development and progress in the field of target detection and have gradually improved and optimized their respective performance and characteristics. YOLOv5 has received widespread attention for its lightweight and efficient performance. It optimizes the network structure, improves operation speed and memory utilization, and excels in real-time applications and resource-limited scenarios. YOLOv6 combines the segmentation and detection tasks by introducing a hybrid matching strategy, which helps to reduce the occurrence of false detections by combining global and local information but may increase the complexity of the model and computational requirements. YOLOv7 has simple yet powerful features, with a relatively simple architecture that is easy to understand and implement, and offers advantages in terms of accuracy and speed of operation. YOLOv8 also introduces a hybrid matching strategy to further improve accuracy, which shares many similarities with YOLOv7 but differs in some key aspects, with YOLOv8’s architecture potentially being more complex and requiring more computational resources. The performance of different YOLO algorithms may vary in different scenarios. It is not necessary that the latest YOLO algorithm is the best choice, and it is necessary to consider the scenarios, resource constraints, speed and accuracy, and other needs to determine the most appropriate algorithm version.

The YOLO series of target detection models are sufficient for weed detection tasks in field environments. Therefore, this study constructed a weed detection model based on YOLOv7 and enhanced the model performance by improving the model, enhancing the image dataset and other operations. The specific research ideas are as follows: We designed four levels of herbicide reduction spraying experiments, utilized UAV to obtain image information of soybean fields, performed weed detection based on the improved YOLOv7 model to determine the optimal level of herbicide application, and examined soybean growth status by obtaining soybean leaf area and dry matter weight at different growth stages.

## 2 Materials and methods

### 2.1 Experimental site

**Figure 1:**
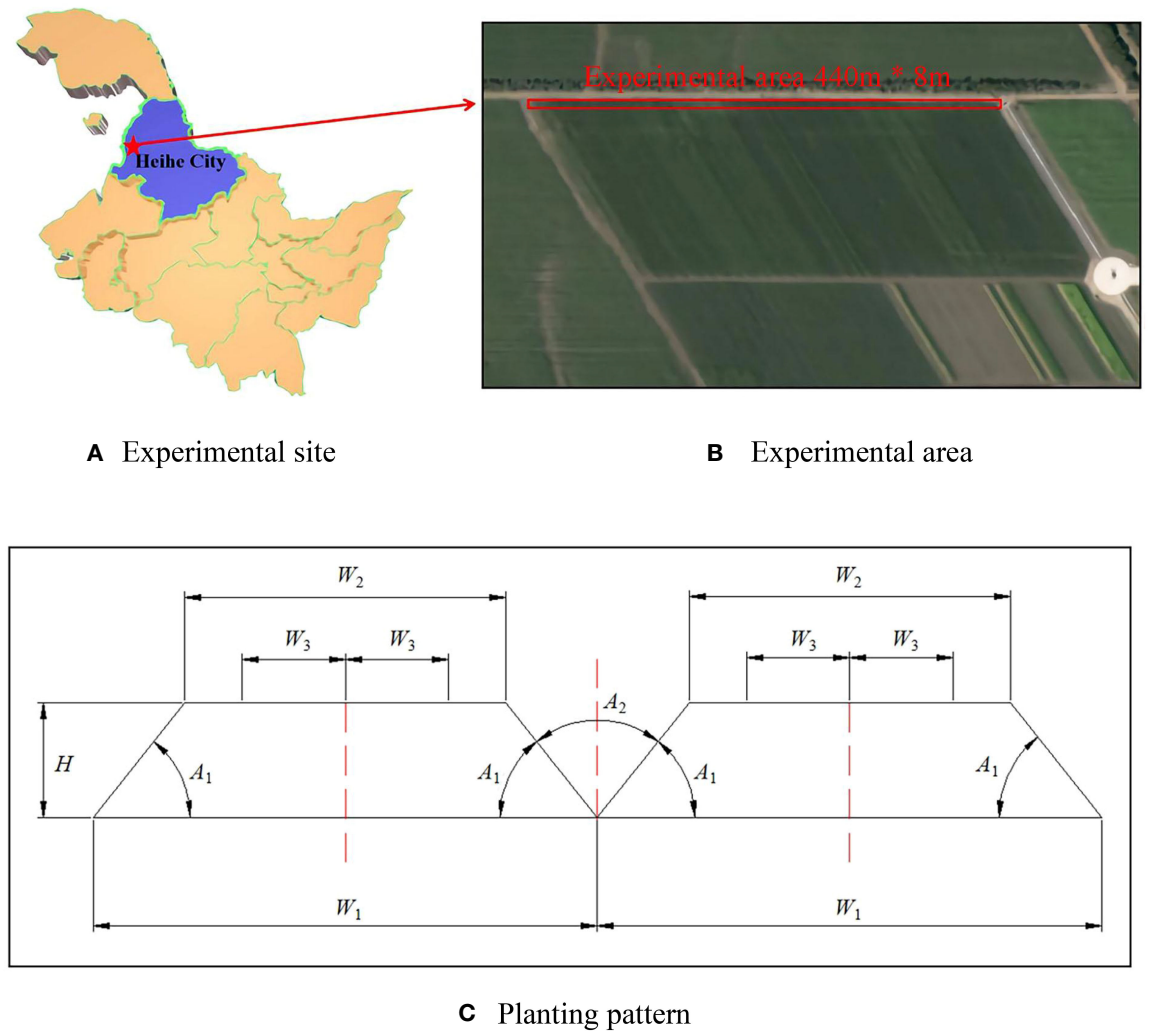
The schematic diagram of the experimental site and planting pattern. **(A)** Experimental site. **(B)** Experimental area. **(C)** Planting pattern.

The experiment was conducted in May–June 2023 at Jianshan Farm (48°86′22''N, 125°36′43''E), Jiusan Reclamation District, Heihe City, Heilongjiang Province, China. Jiusan is the soybean capital of China. Black soil is a fertile and nutrient-rich soil type, and the region’s rich black soil and light-rich climate conditions provide good conditions for soybean growth. The region’s soybean varieties are excellent, and using scientific growing methods, the soybeans here are leading in yield and quality. The experimental area was 440 m × 8 m and the soybean variety was Longken 306. The planting pattern was three rows on a 1.1-m ridge, which fully utilized the marginal effect of the ridge and increased the yield by increasing the number of seedling holding plants per unit area through dense cultivation (Chen et al., 2018). The schematic diagram of the experimental site and planting pattern is shown in **Figure 1**.

As shown in **Figure 1C**, the overall width of a soybean ridge *W*_1_ is 110 cm, the width of the ridge platform *W*_2_ is 70 cm, and the distance between each two rows of soybeans on the platform *W*_3_ is 22.5 cm. The height of the ridge platform to the ground *H* is 25 cm, which is to minimize water evaporation. The angle formed between the ridge platform and the ground *A*_1_ is 51.34°, and the angle formed between the two ridges *A*_2_ is 77.32°, which are designed to increase the light area and ventilation capacity of the ground.

### 2.2 The soybean field weeding experiments

In soybean fields, weeds have a strong growth rate and reproduction capacity and can dominate soybean fields if not controlled in a timely manner. Therefore, it is necessary to choose the right herbicide and dosage to improve the control effect, and this choice is based on a solid scientific basis. Herbicide spraying operations require years of testing and application to prove that the use of combinations of these herbicides is the most suitable for weeds in specific plots, aiming at optimal crop growth and effective weed control.

The field experiments were divided into two parts: one was carried out after the soybean was sown until the emergence of seedlings. This experiment used a soil treatment with herbicides to seal out weeds that were about to emerge and was called the pre-emergence weeding experiment. The second was to use herbicides to spray directly on the stems and leaves of weeds between the emergence of soybeans and the first compound leaf, so as to kill the weeds by inhibiting photosynthesis, which was called the post-emergence weeding experiment. The pre-emergence weeding experiment was conducted at four levels: conventional herbicide spray treatment, electrostatic + reduction of 30%, electrostatic + reduction of 20%, and electrostatic + reduction of 10%. The conventional herbicide spray treatment used the optimal herbicide combinations derived from years of operations at Jianshan Farm (water + acetochlor + thifensulfuron-methyl). When water was 20 L, 150 mL of acetochlor and 4 g of thifensulfuron-methyl were added. The herbicide combination used in the post-emergence weeding experiment was water + fomesafen + thifensulfuron-methyl + bentazone. Based on the results of the four levels of the pre-emergence weeding experiment, the post-emergence weeding experiment was conducted after the dosage of each herbicide was determined using the optimal levels.

Both groups of experiments used backpack sprayer to carry out herbicide spraying operations: the forward speed of the sprayer was 8 km/h, the spray width was 8 m, and the spraying was carried out on seven rows in one operation, with a spray pressure of 0.4 MPa. One experimental area was delineated for each level, with each area measuring 800 m^2^ (100 m long and 8 m wide), and each experimental level was spaced with 10 m.

### 2.3 Research methods

#### 2.3.1 Image dataset preparation

**Figure 2:**
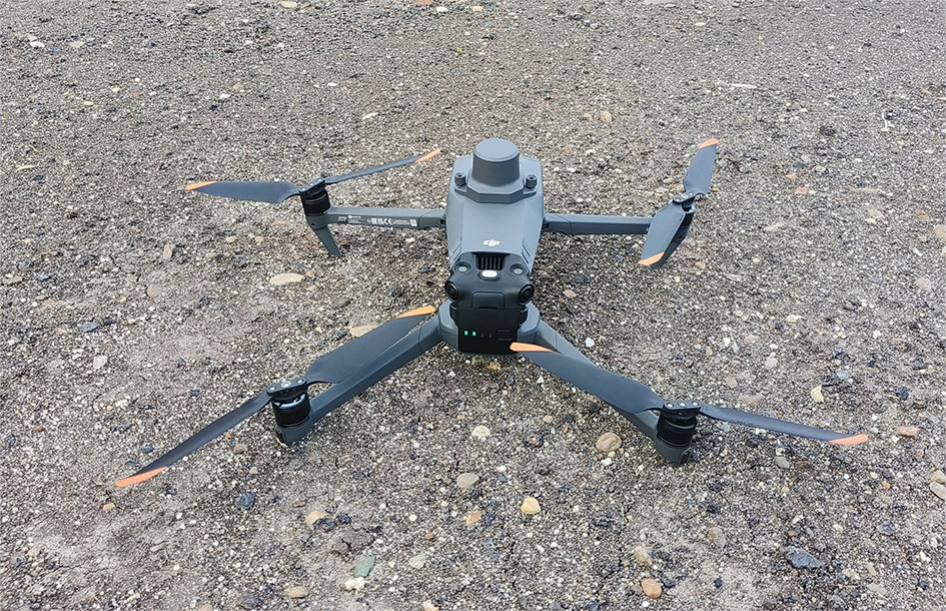
DJI Mavic 3M UAV.

A DJI Mavic 3M UAV as shown in **Figure 2** was utilized to acquire RGB images at soybean emergence (VE), cotyledon (VC), and first node (V1) stages, with an image resolution of 5,280 × 3,956 pixels. RGB sensors are capable of capturing high-resolution images and are suitable for large-scale applications in agricultural production by analyzing details and features in the images (Zhang and Zhao, 2023).

In field environments, UAV images captured under different lighting and weather conditions often have a significant impact on the results of weed detection. On sunny days with sufficient light, the color and texture characteristics of plants are obvious, and they contrast significantly with the surrounding environment. However, on cloudy and rainy days with insufficient lighting, the image quality captured by digital devices will significantly decrease, resulting in unclear edges of the target object, color distortion, lack of texture features, etc., which will affect the detection results. Therefore, in order to better adapt the model to the real field environment and reduce the impact of complex background interference on detection results, image construction datasets were obtained in various environments such as sunny, cloudy, rainy, and strong light, including 500 images.

In our study, the YOLO algorithm was utilized to construct a weed detection model. The model has a large number of parameters; if the dataset size is small, it may cause overfitting and the model cannot converge, thereby affecting detection performance. Therefore, sufficient data are needed to train the model to achieve optimal results. Image enhancement can achieve data augmentation, allowing limited original images to be transformed to increase data diversity and maximize the value of the data. The image enhancement methods we used include horizontal mirroring, brightness enhancement, contrast enhancement, and random flipping of the original image, expanding the number of images to 3,000.

Image annotation is a crucial process, and the accuracy and quality of annotation directly affect the training effectiveness of the model and the accuracy of object detection. We used the image annotation tool LabelImg to annotate weeds in.xml format and stored the annotated images in PascalVOC format. The PascalVOC format contains rich label information, such as categories, target positions, and image sizes. During the labeling process, we set the label of the detected weed target as “weed.” The training set, testing set, and validation set were divided according to the ratio of 8:1:1. The training set is used to train the model, the testing set is used to evaluate the performance of the model and tune it, and the validation set is used to adjust the hyperparameters of the model.

#### 2.3.2 Construction of the weed detection model

**Figure 3:**
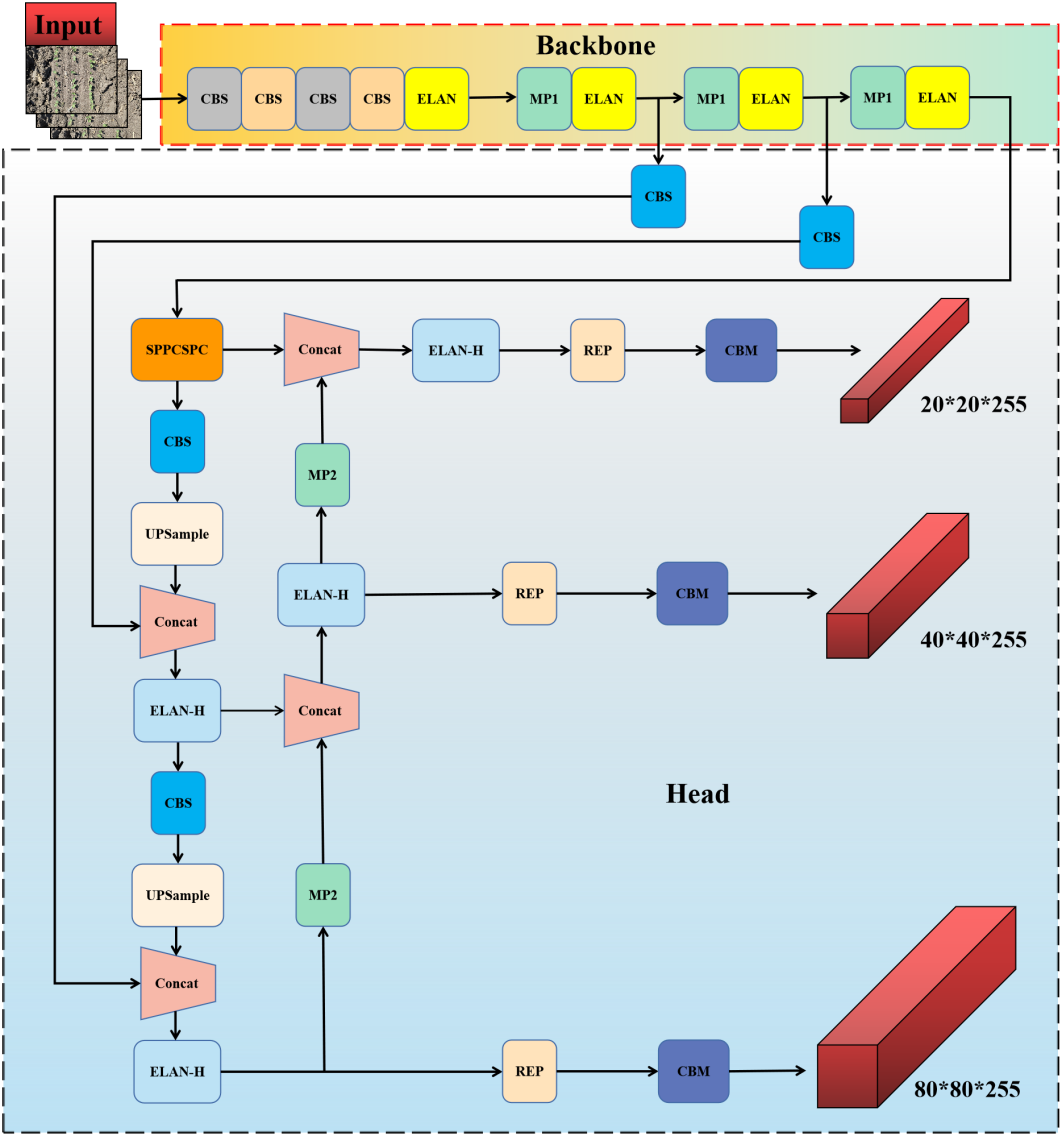
Schematic of the YOLOv7-based weed detection model.

YOLOv7 is a continuous improvement of the previous YOLO series, with accuracy and speed surpassing other YOLO series algorithms on the MSCOCO dataset, achieving a better balance between detection speed and accuracy (Zhu et al., 2023). Therefore, YOLOv7 was used in this study to construct the weed detection model, and we compared its performance in detecting weeds with YOLOv5, YOLOv6, and YOLOv8. The schematic diagram of the weed detection model based on YOLOv7 is shown in **Figure 3**.

**Figure 4:**
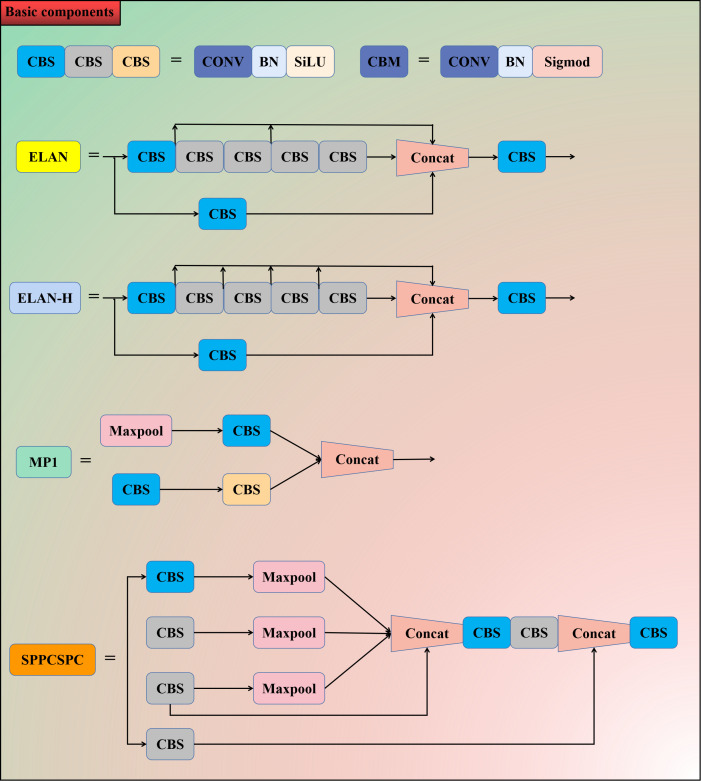
Schematic diagram of each module structure.

The model structure mainly includes the input layer, backbone layer, and head layer. The input layer preprocesses the input image and scales the image to a uniform size in order to meet the training requirements of the backbone network. The backbone layer consists of a number of CBS convolutional modules, ELAN modules, and MP modules. The CBS module consists of a convolutional layer, a batch normalization layer, and SiLU activation function. The ELAN module consists of a number of convolutional modules, which allows for more efficient learning and convergence. The MP module consists of a maximal pooling layer with a number of convolutional modules, which is able to improve the model’s feature extraction capability and detection efficiency. The head layer performs multiscale feature fusion through the path aggregation feature pyramid network (PAFPN) structure. At the prediction end, the REP module is used to adjust the number of image channels of the output features of different scale sizes into bounding boxes, categories, and confidence information, and then the convolutional layer is used as the detection head for downsampling to realize the multiscale detection of large, medium, and small targets. The structure of each module is schematically shown in **Figure 4**.

#### 2.3.3 Improvement and optimization of the weed detection model

**Figure 5:**
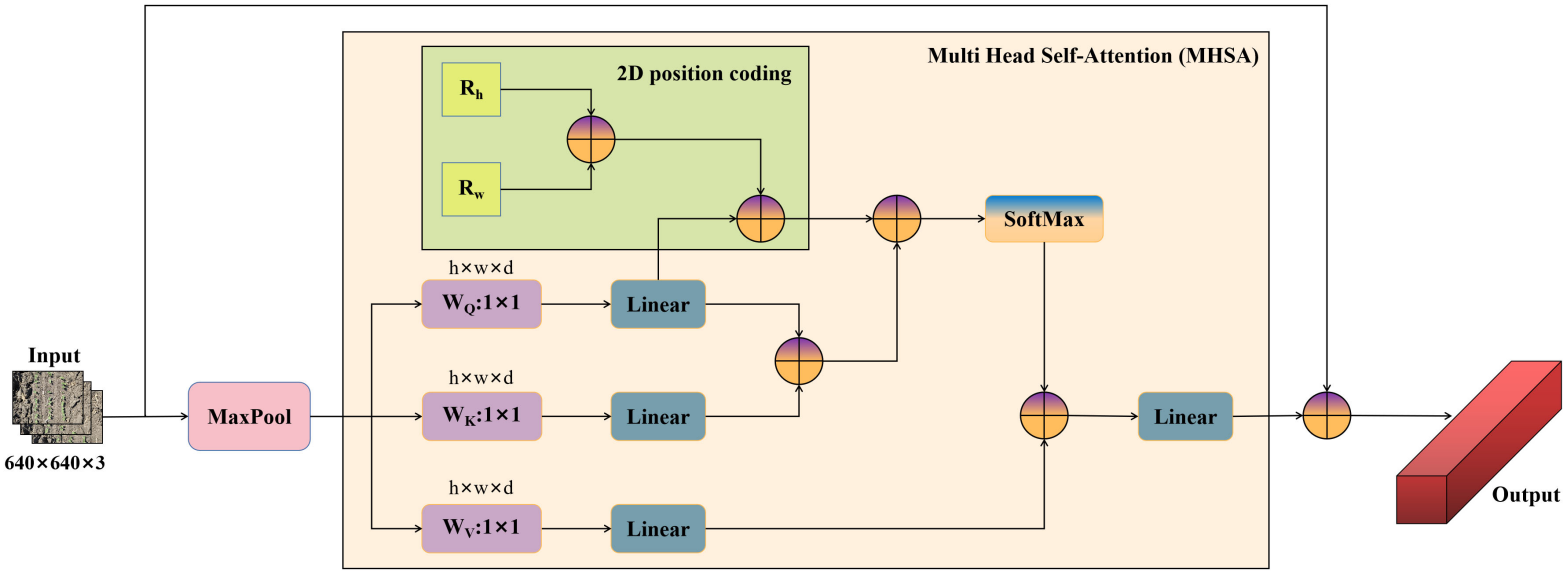
M-MHSA module.

Aiming at the problem of easy leakage of weed targets with few pixels, small coverage area, and little information during target detection, this study proposed YOLOv7-FWeed, a weed detection model based on improved YOLOv7. The model uses F-ReLU as the activation function of the convolutional module in YOLOv7, which can expand the range of the sensory field of the convolutional layer and equip the ordinary convolutional layer with the ability to capture complex visual layouts, thus allowing the convolutional layer to learn more features. It also speeds up model convergence, prevents gradient explosion or gradient vanishing, and solves the spatial insensitivity problem. Another improvement was the addition of the MaxPool multihead self-attention (M-MHSA) module to enhance the network’s learning of global information, which in turn improved the network’s accuracy in recognizing weeds, as shown in **Figure 5** for the M-MHSA module.

The M-MHSA module consists of a maximum pooling layer with a multihead self-attention mechanism, which improves the model learning ability and prevents gradient degradation through the fusion of different feature maps. The input feature maps are first downsampled using the maximum pooling layer, which reduces the feature map size while retaining the main features of weeds and expanding the perceptual field, thus allowing the MHSA module to learn on smaller resolution feature maps. The feature map is then fed into the MHSA module, thus learning a rich hierarchy of associated features across long sequences. This module not only improves the feature extraction ability of the model in complex backgrounds but also avoids the disadvantage of overly focusing attention on itself, and it has a stronger feature representation ability with the same amount of computation.

#### 2.3.4 Evaluation of crop growth after herbicide spraying

If the dosage is too low to achieve effective weed control, uncontrolled weed will continue to grow and reproduce, leading to losses in soybean yield and quality. Therefore, it is necessary to comprehensively evaluate the effectiveness of herbicide reduction spraying based on the growth of weeds and soybeans.

**Figure 6:**
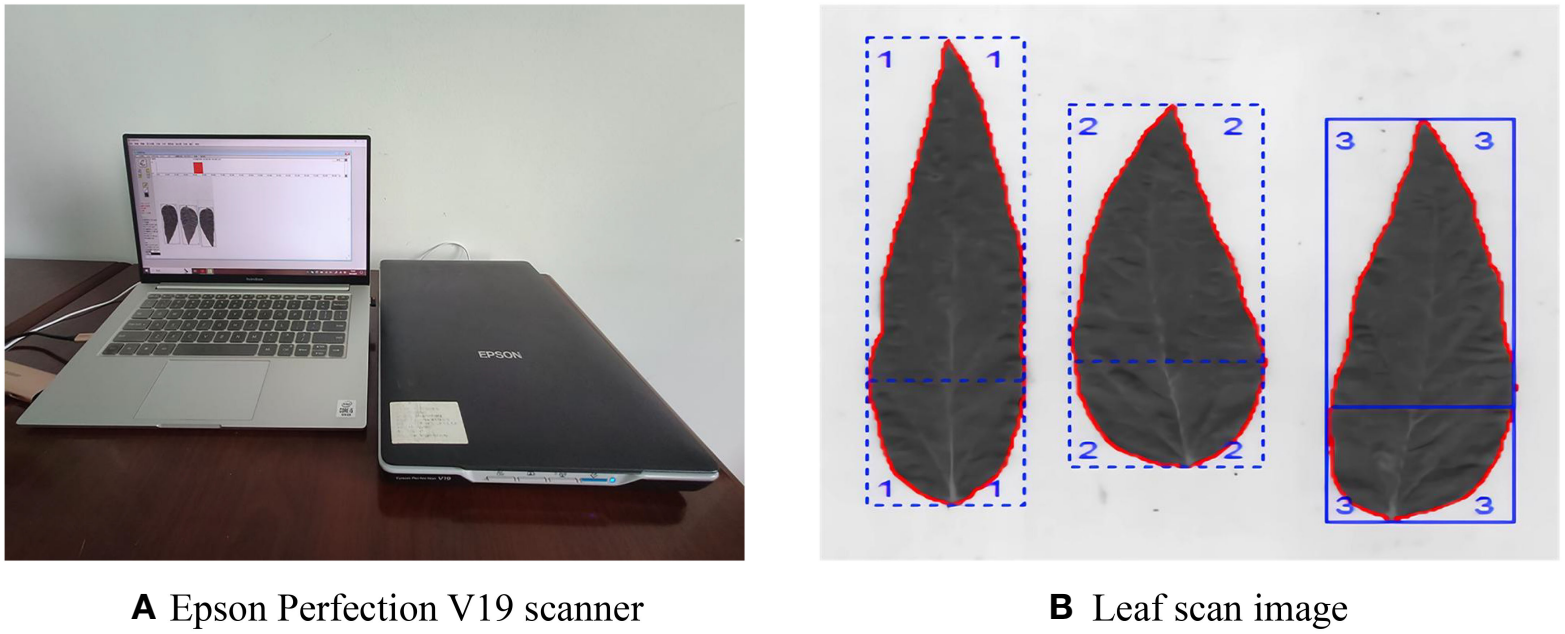
Epson Perfection V19 scanner and scan image. **(A)** Epson Perfection V19 scanner. **(B)** Leaf scan image.

Five sample points were selected in each experimental plot according to the five-point sampling method, each point was approximately 1 m^2^ (0.91 m × 1.1 m), and the number of weeds in the sample points was recorded. The accumulation of physiological metabolites in plants can reflect their growth and development status. During the growth process of weeds, they compete with soybeans for nutrients, leading to changes in soybean nutrient absorption and physiological indicators. When weeds continue to harm soybeans, the changes in soybean leaf area and dry matter weight will decrease with the increase of harm time. Six consecutive plants with consistent growth were selected in each plot during the soybean R1 to R5 stages, and the soybean leaf images were analyzed using an Epson Perfection V19 scanner (Epson Co., Ltd., China) to measure the length and width of their leaf blades and calculate the leaf area, as shown in **Figure 6**. The plants were dried using a WGL-230B electric blast drying oven (Tianjin Tester Instrument Co., Ltd., China), and the dry matter weight of the plants was obtained using an electronic balance.

### 2.4 Evaluation indicators and platform configuration

To evaluate the performance of the YOLOv7 model for weed detection, precision (*P*), recall (*R*), *F*1, and the *mAP* were used as evaluation metrics. *P*, *R*, and *F*1 are calculated as shown in Equation (1–3).


(1) $$P=\frac{T_{P}}{T_{P}+F_{P}}$$

(2) $$R=\frac{T_{P}}{T_{P+}F_{N}}$$

(3) $$F1=\frac{2PR}{P+R}$$

Where *T_P_* represents the number of samples that were correctly predicted as positive, *F_P_* represents the number of negative samples that were predicted as positive, and *F_N_* represents the number of positive samples that were predicted as negative.

The *mAP* is the average of the *AP* of all categories, average precision (*AP*) is the average precision of individual categories, *AP* can reflect the accuracy of the prediction of each category, and *mAP* is used to reflect the accuracy of the whole model. The formulas for the calculation of *AP* and *mAP* are shown in Equation (4, 5).


(4) $$AP=\int_{O}^{1\int dr}\;Precision\times Recall$$

(5) $$mAP=\frac{\sum_{j=1}^{s}AP\left(j\right)}{S}$$

Where *r* is the integral variable, which is the integral of the product of recall and precision; *S* is the number of all categories, and only weeds were detected in the study, so *S* = 1.

The coefficient of determination (*R* ^2^), root mean square error (*RMSE*), and mean absolute error (*MAE*) were used to analyze the differences between the number of predicted weeds and true weeds. *R* ^2^ represents the degree of fitting of the trend line, which can reflect the degree of fitting between the number of weeds of model prediction and manually measured. *RMSE* represents the degree of error dispersion between model prediction and manually measured, representing the stability of the algorithm. *MAE* is the average error between model prediction and manually measured, indicating the accuracy of the algorithm. *R* ^2^, *RMSE*, and *MAE* are calculated as shown in Equation (6–8).


(6) $$R^{2}=\frac{\sum^{}i\left(y_{ip}-y_{m}\right)}{\sum^{}i\left(y_{it}-y_{m}\right)}$$

(7) $$RMSE=\sqrt{\frac{\sum^{}i{\left(y_{ip}-y_{it}\right)}^{2}}{N}}$$

(8) $$MAE=\frac{\sum^{}i\left|y_{ip}-y_{it}\right|}{N}$$

Where *i* is the *i*-th monitoring site, the range of *i* is 1–50, and the summation limit is 50. *y_it_* is the number of true weeds in the *i*-th monitoring site, *y_ip_* is the number of predicted weeds in the *i*-th monitoring site, *y_m_* is the mean value of the number of weeds in each monitoring site, and *N* is the number of monitoring points.

To meet the training requirements for deep learning, the computer environment was configured as follows: an NVIDIA GeForce RTX3080 Ti graphics card with 12 GB of video memory; the CPU was a 9-core Intel(R) Xeon(R) CPU E5-2686 V4 with 32 GB of RAM running on it; and the operating system was Linux, using Pytorch 1.13, Python 3.8, and Cuda 11.6.

## 3 Results

### 3.1 Experimental results of different target detection models

In this study, weed detection was performed using YOLOv5, YOLOv6, YOLOv7, and YOLOv8 models, respectively. The batch size of the model was set to 8 and the number of iterations was 500. The comparison of the experimental results for different target detection models is shown in **Table 1**.

**Table 1:** Comparison of experimental results for different target detection models.

Model	Evaluation indicators				
	*P*	*R*	*F*1	*mAP*	Time/s
YOLOv5	0.7896	0.8174	0.8033	0.8706	0.0432
YOLOv6	0.8427	0.8569	0.8497	0.8817	0.0573
YOLOv7	0.8525	0.8642	0.8583	0.8926	0.0475
YOLOv8	0.8245	0.8298	0.8271	0.8732	0.0512

As can be seen from **Table 1**, within the same number of iterations, the *P*, *R*, *F*1, and *mAP* of the YOLOv7 model were higher than those of the other models, and the detection time for a single image was 0.0475 s, which was slightly lower than that of YOLOv5. YOLOv5 had a slight advantage in detection time, which was mainly due to the relatively lightweight network structure, but it was obviously lower than the other models in other indexes. YOLOv8, the newest model in the series, also achieved good results in detection performance, but due to its relatively complex network structure, in addition to its inferiority to YOLOv7 in other metrics, it additionally increased the detection time. Although the detection time of YOLOv7 was not the shortest, it was able to meet the needs of weed detection in soybean fields. Therefore, YOLOv7 was chosen as the weed detection model for this study and was improved and optimized on this basis to enhance the detection performance in the field environment.

### 3.2 Analysis of weed growth at different herbicide reduction spray levels

**Figure 7:**
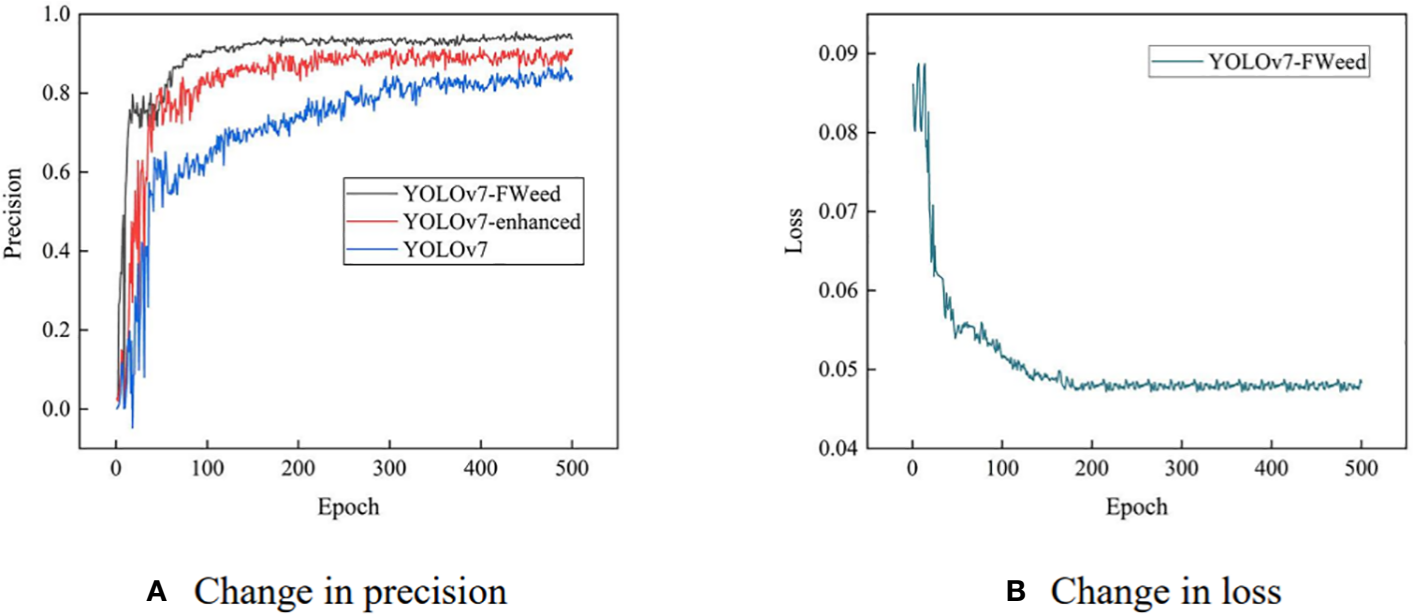
Model precision and loss test results. **(A)** Change in precision. **(B)** Change in loss.

In order to evaluate whether image enhancement can bring substantial improvement to the performance of the model, YOLOv7 was utilized to compare the test accuracy on the original image dataset (500 images) and the enhanced image dataset (3,000 images), which were named YOLOv7 and YOLOv7-enhanced, respectively. In this study, YOLOv7 was improved by using the F-ReLU activation function and adding the M-MHSA module to obtain the YOLOv7-FWeed model, which was utilized to train and test the enhanced image dataset. The official YOLOv7.pt pretraining weights were used in model training; YOLOv7, YOLOv7-enhanced, and YOLOv7-FWeed were utilized to train and test the weed dataset, respectively; and the experimental results of the model precision and loss were obtained as shown in **Figure 7**.

As shown in **Figure 7A**, the fluctuation amplitude of YOLOv7-enhanced was smooth in comparison with YOLOv7. The image enhancement has a certain effect on the model stability enhancement: it improves the image quality, enriches the amount of information, and strengthens the image interpretation and recognition effect. YOLOv7-FWeed achieved superior results in terms of precision compared with the previous two models. The loss curves during 500 epoch were plotted, as shown in **Figure 7B**. The precision and loss of the YOLOv7-FWeed model leveled off approximately 200 epoch, confirming that 500 epoch of training was sufficient, further demonstrating the usefulness of the improved method proposed in this study for weed detection.

There is a balance between precision and recall that needs to be weighed when adjusting model settings. If the model is too sensitive, it may result in an increased error rate, misidentifying non-weed targets as weeds. In addition to the above two metrics, the YOLOv7, YOLOv7-enhanced, and YOLOv7-FWeed models were compared in *R*, *F*1, and *mAP*, and the experimental results are shown in **Table 2**.

**Table 2:** Comparison of experimental results.

Model	Evaluation indicators			
	*P*	*R*	*F*1	*mAP*
YOLOv7	0.8525	0.8642	0.8583	0.8926
YOLOv7-enhanced	0.8865	0.8980	0.8922	0.9347
YOLOv7-FWeed	0.9496	0.9125	0.9307	0.9662

**Figure 8:**
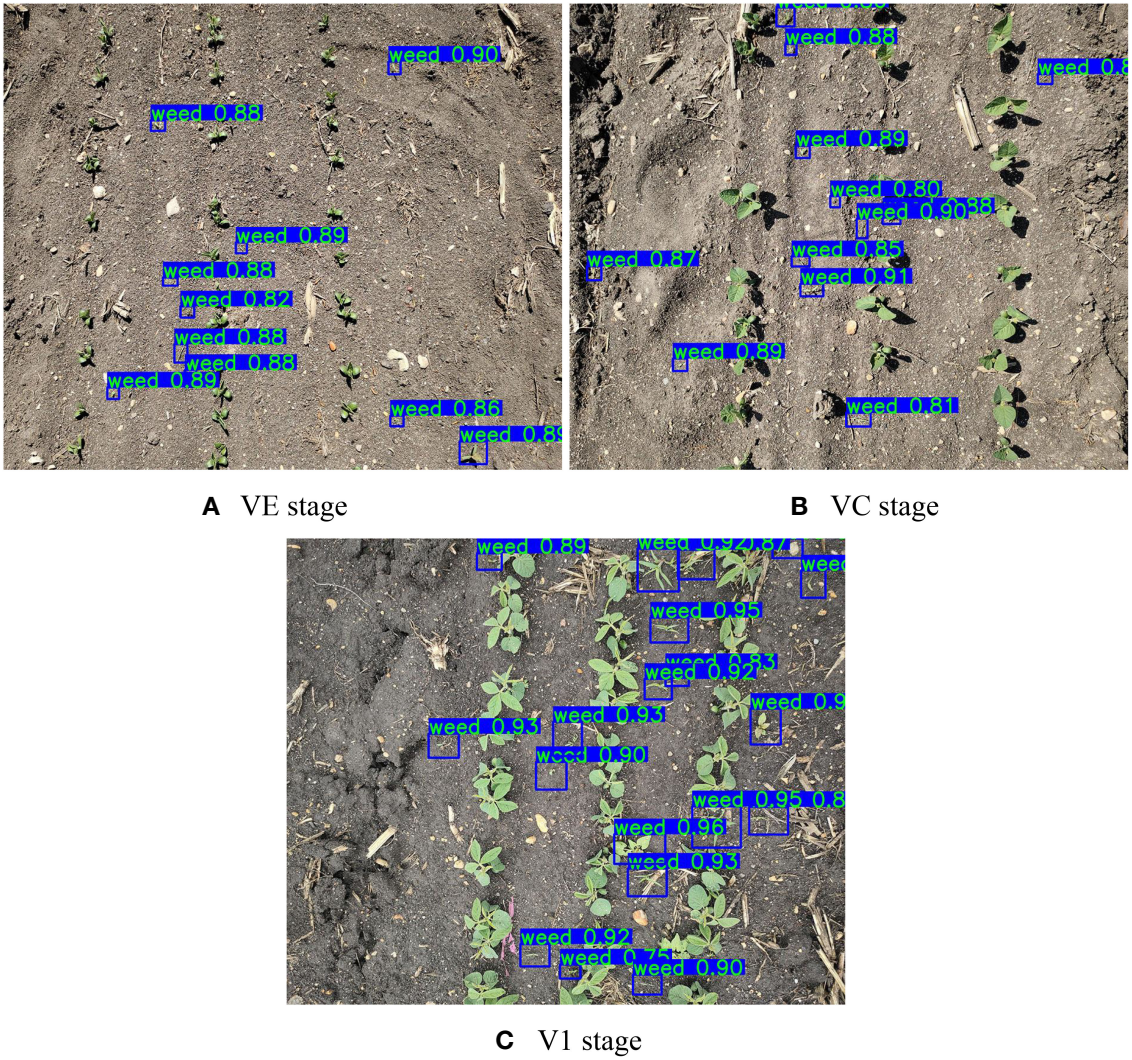
Results of weed detection at different growth stages in soybean. **(A)** VE stage. **(B)** VC stage. **(C)** V1 stage.

Overall, YOLOv7-FWeed achieved the best results in all the metrics. The *P* was 0.9496, the *R* was 0.9125, the *F*1 was 0.9307, and the *mAP* was 0.9662. All the metrics were improved by 0.0483–0.0971 compared with the original model of YOLOv7. These results proved the effectiveness of YOLOv7-FWeed for weed detection. This model achieved detection by dividing the image into grids and predicting whether each grid contains weeds. After the model has completed training and testing, field images were acquired and input to the model for validation, and the model assigned detection frames to the detected weed targets and gave a confidence level for each detection frame. Confidence is a quantitative metric that indicates how certain the model is about the targets included in a given detection frame. The results of applying the YOLOv7-FWeed model to detect weeds at different stages of soybean growth are shown in **Figure 8**.

**Figure 9:**
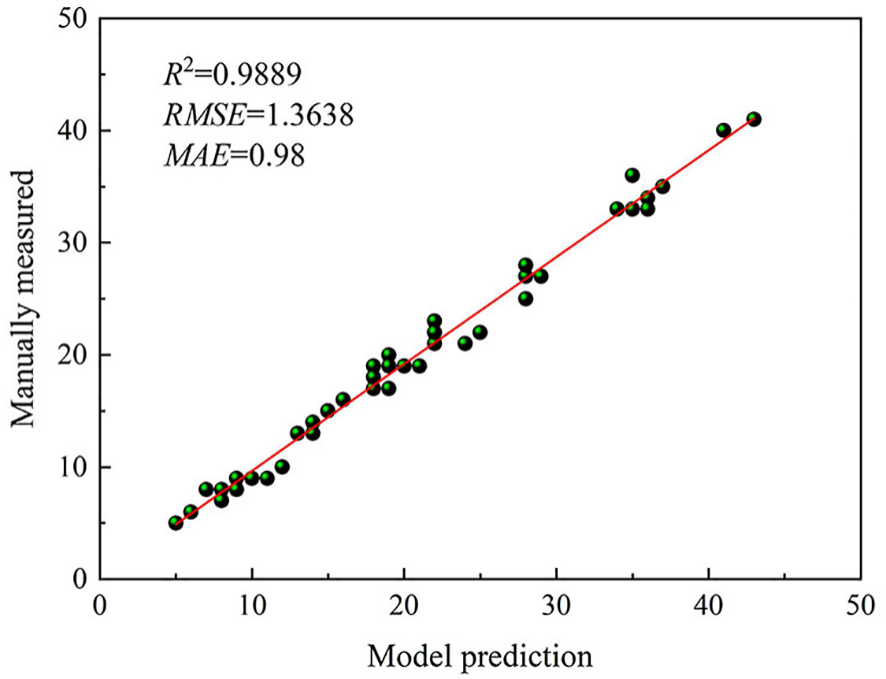
Fit effects of model prediction and manually measured.

The area of the plots covered in the figure was approximately 1 m^2^, and in this study, it was necessary to capture as many weed targets to be detected as possible, as long as the weeds were selected by the frame, which could be regarded as a valid result whether the confidence level was high or low. It could be seen that the weeds could be accurately selected in different growth stages, and basically, no weeds were missed, indicating that the model learned the real characteristics of the weed targets, and the recognition effect was satisfactory. To further evaluate the model performance, images of 50 points were acquired in the field to obtain the number of weeds detected using the model and manually measured, respectively. The analysis was carried out using Equations (6)–(8) and a linear relationship graph was obtained as shown in **Figure 9**.

As can be seen in **Figure 9**, *R* ^2^ was 0.9889, indicating a good fit of the model and a strong correlation between the number of predicted and true weeds. *RMSE* was 1.3638 and *MAE* was 0.98. The values of *RMSE* and *MAE* were small, indicating that the model predicted better.

### 3.3 Analysis of weed growth at different herbicide reduction spray levels

#### 3.3.1 Results of the pre-emergence weeding experiment

The number of weeds was monitored during May 23–June 11, which was from the beginning of soybean emergence until the seedling weeding experiment, and the number of weeds statistics was obtained as shown in **Table 3**.

**Table 3:** The number of weeds at different points.

Date	Sampling point location	Number of weeds			
		Level 1	Level 2	Level 3	Level 4
5.23	1	10	3	3	2
	2	5	1	4	1
	3	14	3	5	2
	4	9	24	2	6
	5	3	5	1	1
	**Total number**	**41**	**36**	**15**	**12**
5.27	1	35	17	8	15
	2	14	8	5	14
	3	39	15	16	16
	4	30	34	42	23
	5	4	17	25	4
	**Total number**	**122**	**91**	**96**	**72**
5.29	1	57	31	18	20
	2	19	14	12	21
	3	64	22	38	21
	4	45	82	62	42
	5	7	26	38	9
	**Total number**	**192**	**175**	**168**	**113**
6.1	1	72	40	23	20
	2	30	20	15	26
	3	79	23	48	25
	4	64	91	73	56
	5	9	37	42	13
	**Total number**	**254**	**211**	**201**	**140**
6.4	1	75	58	34	25
	2	36	28	19	30
	3	99	38	62	31
	4	77	108	93	69
	5	11	49	65	18
	**Total number**	**298**	**281**	**273**	**173**
6.7	1	84	63	38	40
	2	41	30	23	38
	3	116	42	66	41
	4	81	121	104	81
	5	12	62	70	25
	**Total number**	**334**	**318**	**301**	**225**
6.11	1	89	66	40	47
	2	48	31	26	42
	3	123	44	71	49
	4	84	129	108	92
	5	13	70	75	32
	**Total number**	**357**	**340**	**320**	**262**

Level 1 is the conventional herbicide spray treatment, level 2 is the electrostatic + reduction of 30%, level 3 is the electrostatic + reduction of 20%, and level 4 is the electrostatic + reduction of 10%. As can be seen in **Table 3**, on May 23, the number of weeds in the field ranged from 1 to 24/m², with most of them being less than 10/m². Over time, the number of weeds gradually increased, and by June 1, each point had a mean value greater than 40/m². After that, on June 7 and June 11, the number of weeds was greater than 50/m² and 60/m².

**Figure 10:**
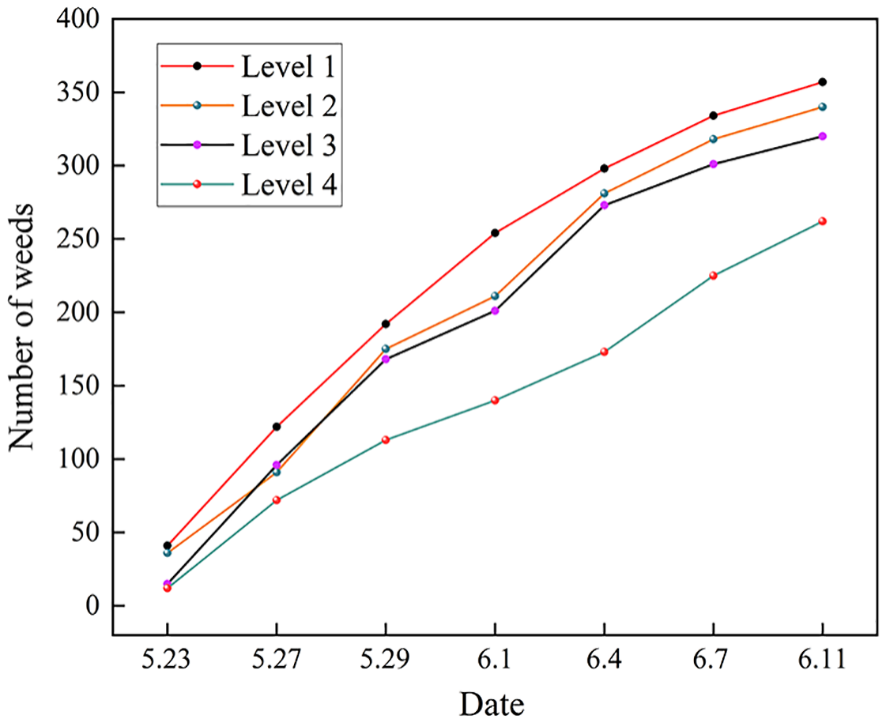
Changes in the number of weeds at different levels.

Based on the total number of weeds at different herbicide reduction spray levels on different dates in **Table 3**, changes in the number of weeds were plotted as shown in **Figure 10**.

The trend of the total number of weeds over time for different herbicide reduction spraying levels can be further observed from **Figure 10**. It can be seen that during May 23–June 4, the number of weeds of level 1, level 2, and level 3 showed a rapid growth trend; after June 4, the number of weeds grew at a relatively slow rate. Level 4, on the other hand, showed a trend of slow growth in the number of weeds throughout the cycle, and the total number of weeds at this level has been at a minimum.

When conducting reduced herbicide spraying experiments, the dosage is too low to achieve effective weed control, and uncontrolled weeds will continue to grow and multiply, leading to losses such as reduced crop yields and poorer quality. When the application dose exceeds the recommended level, chemical pesticides may remain in the soil, water sources, and crops, with negative impacts on the ecosystem. Therefore, based on the results of the above analysis, effective herbicide spraying measures need to be taken in controlling weeds in the field, level 4 carried out a reduction of 10% and had a certain inhibitory effect on the growth of weeds, and the level was used in subsequent experiments to observe whether the number of weeds was effectively controlled.

#### 3.3.2 Results of the post-emergence weeding experiment

The post-emergence weeding experiment was conducted on June 13, which was during the V2 and V3 stages of soybean, a critical time for field management for chemical herbicide spraying. Herbicide spraying was conducted on the four previous experimental areas using the electrostatic + reduction of 10%. When water was 120 L, fomesafen was 0.9 L, bentazone was 1.5 L, and thifensulfuron-methyl was 20 g. Changes in the number of weeds were monitored at 2, 6, and 9 days after spraying, and the results are shown in **Table 4**.

**Table 4:** Changes in the number of weeds.

Date	Sampling point location	Number of weeds after electrostatic spraying + herbicide reduction of 10% treatment			
		Level 1	Level 2	Level 3	Level 4
6.15	1	72	49	27	34
	2	35	20	21	31
	3	95	38	59	35
	4	70	106	94	72
	5	6	34	65	25
	**Total number**	**278**	**247**	**266**	**197**
	**Reduced number**	**79**	**93**	**54**	**65**
6.19	1	29	7	8	12
	2	12	8	5	18
	3	18	7	14	9
	4	58	14	15	35
	5	2	12	6	6
	**Total number**	**119**	**48**	**48**	**80**
	**Reduced number**	**159**	**199**	**218**	**117**
6.22	1	22	7	8	10
	2	10	7	5	15
	3	16	6	14	8
	4	5	12	13	24
	5	2	12	6	6
	**Total number**	**55**	**44**	**46**	**63**
	**Reduced number**	**64**	**4**	**2**	**17**

As can be seen from **Table 4**, 2 days after spraying, the number of weeds showed a slow decreasing trend; 6 days after spraying, the number of weeds showed a sharp decreasing trend; 9 days after spraying, the decreasing trend of the number of weeds tended to level off, and the number of weeds was effectively controlled. On June 22, five points were selected for sampling in the plots where the conventional herbicide spray treatment was carried out, and the total number of weeds was 41. Compared with the four experimental areas treated with the electrostatic + reduction of 10%, the number of weeds in the four experimental areas was slightly higher than that in the plots where the conventional herbicide spray treatment was carried out. The results showed that the experimental protocol utilizing the electrostatic + reduction of 10% was feasible and effective in using less herbicide, but it was not clear if the treatment would have an impact on the subsequent growth of soybean. Therefore, after herbicide spraying was conducted, continuous attention was paid to the growth and health of soybean in subsequent stages.

### 3.4 Analysis of soybean growth after herbicide spraying

**Figure 11:**
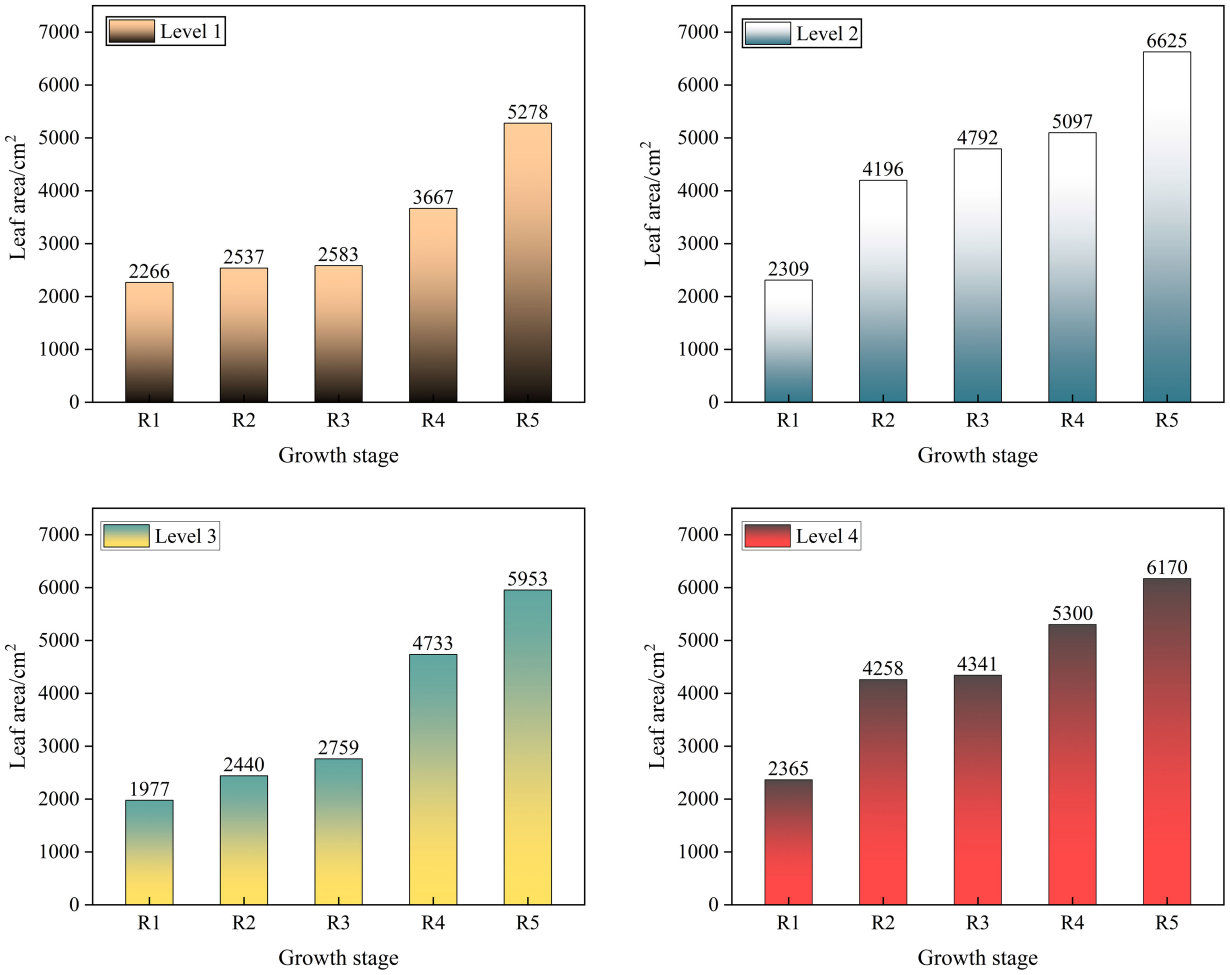
Changes in soybean leaf area.

In this study, subsequent growth was assessed by changes in leaf area and dry matter weight of soybean. Crop leaf area determines the crop’s ability to absorb solar radiation energy for photosynthesis (Chen et al., 2022). Different spray rates of herbicides may reduce the leaf area, and if the leaf area increases after spraying, the soybean is growing well; if the leaf area decreases, there may be a growth problem. The changes in leaf area of soybean at stages R1–R5 are shown in **Figure 11**.

The results showed that at the R1–R5 stages of soybean, the soybean leaf area showed an increasing trend with the continuous change of time, indicating that the electrostatic + reduction of 10% did not hinder the increase of soybean leaf area.

**Figure 12:**
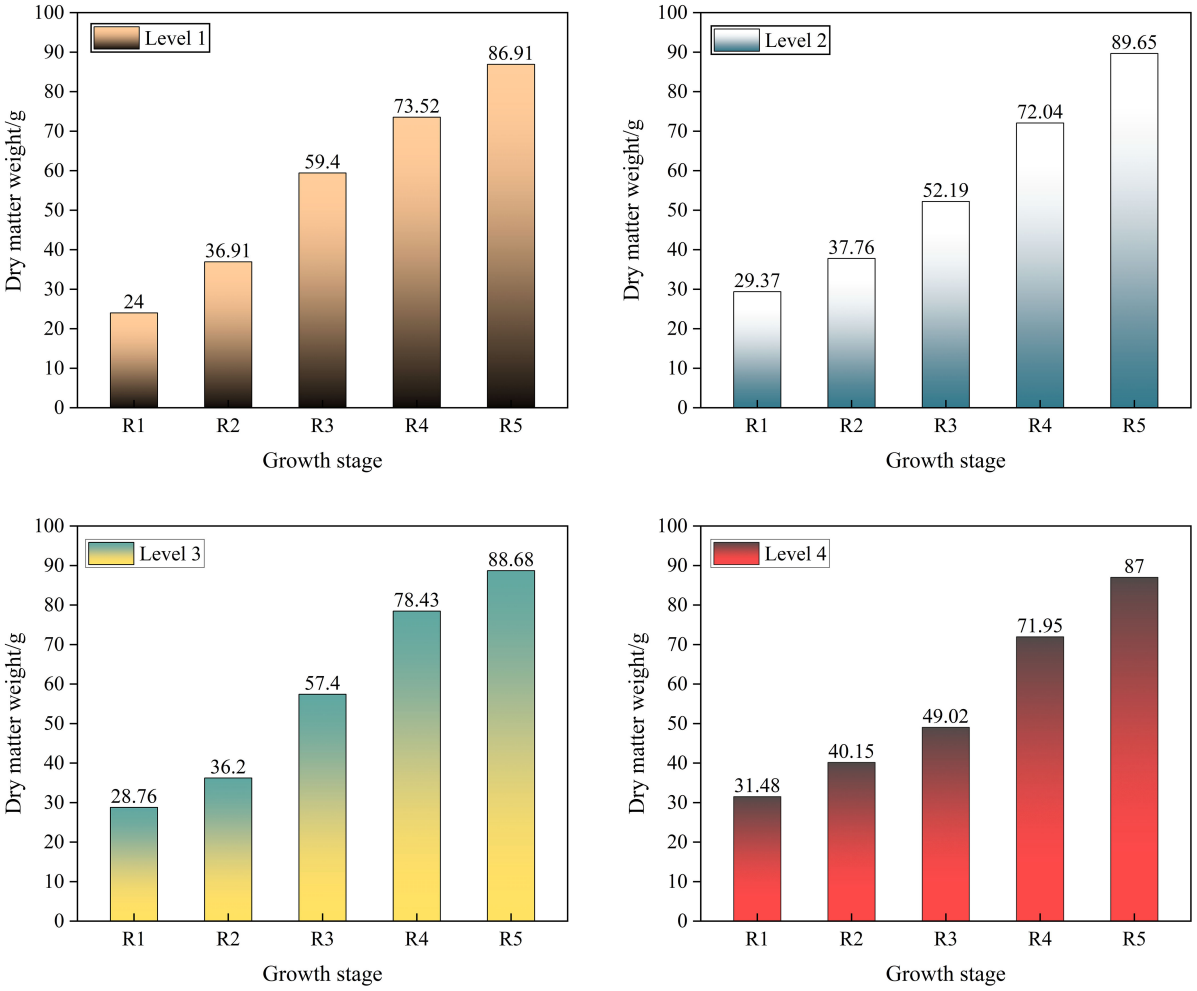
Changes in soybean dry matter weight.

Dry matter weight reflects the true crop growth status because it eliminates the effect of moisture on measurements and is the basis for crop yield formation (Liu et al., 2016). If the dry matter weight increases after spraying, soybean is performing well in terms of overall growth and development; if the dry matter weight decreases, there may be problems such as growth limitation or insufficient nutrient uptake. The changes in dry matter weight of soybean from R1 to R5 stages are shown in **Figure 12**.

The results showed that in the R1–R5 stages of soybean, the dry matter mass of soybean showed an increasing trend with the continuous change of time, indicating that the electrostatic + reduction of 10% does not hinder the accumulation of nutrients in soybean. Taken together, both soybean leaf area and dry matter weight showed an increasing trend, indicating that this spray level can be utilized during the seedling weeding stage of soybean, which can effectively control the growth of weeds on the basis of reduced herbicide spraying and does not impede the growth and development of soybean.

## 4 Discussion

### 4.1 Comparative analysis of related studies

In this study, deep learning was used for weed detection in soybean fields and achieved better results. In a similar weed detection work, this work is very challenging due to the similarity between crops and weeds. To overcome these problems, Wang A. et al. (2022) proposed a pixel-level integrated data enhancement method and the TIA-YOLOv5 network for weed and crop detection in complex field environments, and their *F*1 and *mAP* were 0.7 and 0.9, respectively. We used F-ReLU as the activation function of the convolutional module in YOLOv7 to give the ordinary convolutional layer the ability to capture complex visual layouts, thus allowing the convolutional layer to learn more weed features and improve *F*1 and *mAP*. Liu S. et al. (2022) proposed a maize weed detection model—YOLOv4-tiny—that combines an attentional mechanism and a spatial pyramid pooling structure with a *mAP* of 0.8669. We added the M-MHSA module to enable it to learn on smaller resolution feature maps, which preserved the main features of weeds and improved the accuracy of the model in recognizing weeds.

Although the model proposed in this study is able to recognize weeds in soybean fields, there are still some noteworthy issues that need to be further investigated; for example, the model still misses or incorrectly detects weeds on some small targets, and small target detection is still a challenge. Therefore, we will consider adding appropriate feature fusion algorithms to our model to further improve the model’s ability to recognize weeds. In the future, by combining deep learning with other techniques, we can improve the automatic feature learning and adaptation capabilities of deep learning, so that it can realize its great potential to better solve the problem of weed detection in complex environments.

### 4.2 Application of precision spraying herbicide technology

Weed detection is the basis for conducting future applications of precision spraying herbicide technology, and there is a great need for ongoing research. Detection and identification of weeds in the field using computer vision and data analytics are at the core of precision herbicide spraying in agriculture, and these techniques help the spraying equipment to distinguish between the types of plants, identify weeds, and motivate spraying actions. Precision spraying herbicide technology allows for the application of precise doses of herbicides only on target plants and avoids open soil areas, which can dramatically reduce the cost of herbicide inputs (He, 2022).

Current precision herbicide spraying also faces significant challenges, such as missing the best time to spray and failing to accurately identify weed distribution. The most effective solution is to map and track spray areas by constructing and processing field data and designing and training AI algorithms for crop growth stage monitoring and accurate weed identification. With the amount of data in the field increasing every year, it can be difficult for growers to process it all on their own, which can be simplified by building databases and preparing them for the specific tasks they will be used for. AI-based agricultural management systems can plan spraying at the optimal time when weeds are in the early stages of canopy development and quickly analyze new images to identify weeds and other plants. By collecting field images from UAVs and applying weed recognition algorithms to plan the exact distribution of herbicides on each field at the early stages of weed development, and then generating weed distribution maps, we can accurately monitor weed growth and record weed location information, so as to control spraying equipment to spray accurately.

### 4.3 Applications of satellite remote sensing technology

Satellite remote sensing technology has a wide range of applications in agriculture. Zhang et al. (2021) extracted rice planting area using multitemporal Gaofen 6 wide-format camera remote sensing images of the critical rice season. Song et al. (2022) applied satellite-borne LiDAR data to realize the estimation of forest aboveground biomass. He et al. (2022) estimated the ratio of photosynthetically active radiation absorption and production potential of regional maize based on the resampling of ground-truth canopy hyperspectral reflectance by GF1 satellite. However, limited by the temporal and spatial resolution of data acquisition, satellite remote sensing is more difficult to be applied to small experimental areas and high-frequency dynamic monitoring (Sindhuja et al., 2015), and the satellite revisit cycle is long and subject to greater interference from the atmosphere, clouds, rain, and snow (Yue et al., 2017). However, when faced with the task of continuous monitoring of large plots of land, the endurance of UAVs and the need for long hours of operation bring certain limitations. With the continuous development of satellite remote sensing technology, it provides new technical means for the precise management of crop fields. In the future, we can try to utilize satellite remote sensing to carry out weed detection research, and strive to provide effective solutions for the application of satellite remote sensing technology in the detection of small targets.

### 4.4 Challenges and prospects

In this study, we used leaf area and dry matter weight to evaluate soybean growth after reduced herbicide spraying, and these metrics can provide a basis for the extent to which herbicides affect crop growth and development. However, in the actual assessment, the possible influence of crop varieties, growing environment, weather conditions, and other factors on the results should also be taken into account. In addition, appropriate indicators should be selected for assessment based on the particular herbicide and target weed. The above patterns are trends in general but may vary depending on crop varieties, growing environments, and other factors. In the next study, crop growth can be comprehensively assessed and judged by combining with other factors observed and recorded in the actual field, such as environmental conditions, herbicide doses, crop varieties, and so on.

We have studied weed detection and different herbicide reduction spray levels in soybean fields, but there are still some limitations. The relationship between herbicides and weeds may have long-term and complex effects, and over the course of a year, while we could observe changes in weed growth, we did not take into account other factors that may affect weed abundance and distribution. We need to consider more variables and the effect of data quality on model performance to improve the generalization ability of the model. Finally, we should actively explore the possibility of extending the duration of the experiment, expanding the scope of the experiment, etc., in order to conduct a more comprehensive assessment. Through these measures, we hope to gain a deeper understanding of the number and distribution of weeds in soybean fields, develop more accurate herbicide reduction spraying strategies, and provide a scientific basis for agricultural production.

## 5 Conclusions

Herbicide reduction spraying can be achieved through rational dosing and spraying practices. This study explored the combination of electrostatic spraying technology with reduced herbicide use to achieve the goal, and the effect was reflected by weed detection and subsequent soybean growth. We proposed weed detection via the YOLOv7-FWeed model by adding the M-MHSA module, consisting of a maximum pooling layer with a multihead self-attention mechanism, to the original YOLOv7 model. This module reduced the feature map size while retaining the main features of weeds and expanding the perceptual field, allowing the model to learn on smaller-resolution feature maps. By doing so, the model’s ability to extract features in complex backgrounds was improved, and the drawback of overly focusing attention on itself was also avoided. The model was able to accurately detect weeds at different growth stages, with essentially no missed detections. After spraying soybean utilizing reduced herbicide rates, soybean performed well in terms of overall growth and development, showing a consistent increase in leaf area and dry matter weight. Weeding in soybean fields can be better achieved through the combination of herbicide reduction and electrostatic spraying techniques. In the future, we can explore the incorporation of new modules in the weed detection model to further improve its performance, and by combining more factors to comprehensively assess soybean growth, the weed distribution in the plot can be mapped based on the results of weed detection, laying a theoretical foundation for application of precision spraying herbicide technology. The technology proposed in this study helps to better understand the growth and distribution pattern of weeds in agricultural fields, better manage weeds in agricultural fields, provide a more scientific and reasonable application program, improve the efficiency of pesticide use, provide more comprehensive and accurate information support for on-site diagnostic work, and improve the yield and quality of crops.

## Data availability statement

The original contributions presented in the study are included in the article. Further inquiries can be directed to the corresponding author.

## Author contributions

JL: Conceptualization, Data curation, Investigation, Methodology, Software, Visualization, Writing – original draft. WZ: Resources, Supervision, Writing – review & editing. HZ: Data curation, Visualization, Writing – original draft. CY: Visualization, Writing – original draft. QL: Conceptualization, Investigation, Resources, Supervision, Visualization, Writing – review & editing.

## References

[B1] Ahmad F. Qiu B. Dong X. Ma J. Huang X. Ahmed S.. (2020). Effect of operational parameters of UAV sprayer on spray deposition pattern in target and off-target zones during outer field weed control application. Comput. Electron. Agric. 172, 105350. doi: 10.1016/j.compag.2020.105350

[B2] Chen H. Li T. Wang H. Wang Y. Wang X. (2018). Design and parameter optimization of pneumatic cylinder ridge three-row close-planting seed-metering device for soybean. Trans. Chin. Soc Agric. Eng. 34 (17), 16–24. doi: 10.11975/j.issn.1002-6819.2018.17.003

[B3] Chen J. Wu F. Han Y. Li X. Wang Z. Feng L.. (2022). Nondestructive measurement of cotton leaf area at the seedling stage based on thermal infrared and visible images. Trans. Chin. Soc Agric. Eng. 38 (15), 179–185. doi: 10.11975/j.issn.1002-6819.2022.15.019

[B4] Fang H. Niu M. Xue X. Ji C. (2022). Effects of mechanical-chemical synergistic weeding on weed control in maize field. Trans. Chin. Soc Agric. Eng. 38 (06), 44–51. doi: 10.11975/j.issn.1002-6819.2022.06.005

[B5] Fatima H. S. Hassan I. Hasan S. Khurram M. Stricker D. Afzal,. M. Z. (2023). Formation of a lightweight, deep learning-based weed detection system for a commercial autonomous laser weeding robot. Appl. Sci. 13 (6). doi: 10.3390/app13063997

[B6] Feng A. Zhou J. Vories E. Sudduth K. A. (2020). Evaluation of cotton emergence using UAV-based narrow-band spectral imagery with customized image alignment and stitching algorithms. Remote Sens. 12 (11), 176. doi: 10.3390/rs12111764

[B7] Gong C. Liu Y. Ma Y. Zhan X. Zhou Z. Zhu X.. (2022). Influence of electrostatic spraying on drift and deposition distribution. J. Sichuan Agric. Univ. 40 (02), 220–226 + 242. doi: 10.16036/j.issn.1000-2650202109029

[B8] He J. Guo Y. Zhang Y. Yang X. LIU T. Wang L. (2022). Dynamic estimation FPAR of summer maize based on GF-1 satellite data. Trans. Chin. Soc Agric. Mach. 53 (04), 164–172 + 321. doi: 10.6041/j.issn.1000-1298.2022.04.017

[B9] He X. (2022). Research and development of efficient plant protection equipment and precision spraying technology in China: a review. J. Plant Prot. 49 (01), 389–397. doi: 10.13802/j.cnki.zwbhxb.2022.2022827

[B10] Jiang K. Xie T. Yan R. Wen X. Li D. Jiang H.. (2022). An attention mechanism-improved YOLOv7 object detection algorithm for hemp duck count estimation. Agric. 12 (10). doi: 10.3390/agriculture12101659

[B11] Lan Y. Zhang H. Wen S. Li S. (2018). Analysis and experiment on atomization characteristics and spray deposition of electrostatic nozzle. Trans. Chin. Soc Agric. Mach. 49 (04), 130–139. doi: 10.6041/j.issn.1000-1298.2018.04.015

[B12] Liu M. Gao T. Ma Z. Song Z. Li F. Yan Y. (2022). Target detection model of corn weeds in field environment based on MSRCR algorithm and YOLOv4-tiny. Trans. Chin. Soc Agric. Mach. 53 (02), 246–255 + 335. doi: 10.6041/j.issn.1000-1298.2022.02.026

[B13] Liu S. Jin Y. Ruan Z. Ma Z. Gao R. Su Z. (2022). Real-time detection of seedling maize weeds in sustainable agriculture. Sustain. 14 (22), 15088. doi: 10.3390/su142215088

[B14] Liu B. Li J. He J. Shi Z. (2016). Estimation models of above-ground dry matter accumulation of summer maize based on hyperspectral remote sensing vegetation indexes. Trans. Chin. Soc Agric. Mach. 47 (03), 254–262. doi: 10.6041/j.issn.1000-1298.2016.03.036

[B15] Mohidem N. A. Che’Ya N. N. Juraimi A. S. Llahi W. F. F. Roslim M. H. M. Sulaiman N.. (2021). How can unmanned aerial vehicles be used for detecting weeds in agricultural fields? Agric. 11 (10), 1004. doi: 10.3390/agriculture11101004

[B16] Pei H. Sun Y. Huang H. Zhang W. Sheng J. Zhang Z. (2022). Weed detection in maize fields by UAV images based on crop row preprocessing and improved YOLOv4. Agric. 12 (7), 975. doi: 10.3390/agriculture12070975

[B17] Rai N. Zhang Y. Ram B. G. Schumacher L. Yellavajjala R. K. Bajwa S.. (2023). Applications of deep learning in precision weed management: A review. Comput. Electron. Agric. 206, 107698. doi: 10.1016/j.compag.2023.107698

[B18] Rakhmatulin I. Kamilaris A. Andreasen C. (2021). Deep neural networks to detect weeds from crops in agricultural environments in real-time: A review. Remote Sens. 13 (21). doi: 10.3390/rs13214486

[B19] Ru Y. Jin L. Jia Z. Bao R. Qian X. (2015). Design and experiment on electrostatic spraying system for unmanned aerial vehicle. Trans. Chin. Soc Agric. Eng. 31 (8), 42–47. doi: 10.3969/j.issn.1002-6819.2015.08.007

[B20] Sindhuja S. Khot L. R. Espinoza C. Z. Jarolmasjed S. Sathuvalli V. R. Vandemark G. J.. (2015). Low-altitude, high-resolution aerial imaging systems for row and field crop phenotyping: A review. Eur. J. Agron. 70, 112–123. doi: 10.1016/j.eja.2015.07.004

[B21] Song H. Shu Q. Xi L. Qiu S. Wei Z. Yang Z. (2022). Remote sensing estimation of forest above-ground biomass based on spaceborne lidar ICESat-2/ATLAS data. Trans. Chin. Soc Agric. Eng. 38 (10), 191–199. doi: 10.11975/j.issn.1002-6819.2022.10.023

[B22] Tang Y. Qiu J. Zhang Y. Wu D. Cao Y. Zhao K.. (2023). Optimization strategies of fruit detection to overcome the challenge of unstructured background in field orchard environment: A review. Precis Agric. 24, 1183–1219. doi: 10.1007/s11119-023-10009-9

[B23] Teimouri N. Jørgensen R. N. Green O. (2022). Novel assessment of region-based CNNs for detecting monocot/dicot weeds in dense field environments. Agron. 12 (5). doi: 10.3390/agronomy12051167

[B24] Wang Q. Cheng M. Huang S. Cai Z. Zhang J. Yuan H. (2022). A deep learning approach incorporating YOLOv5 and attention mechanisms for field real-time detection of the invasive weed Solanum rostratum Dunal seedlings. Comput. Electron. Agric. 199, 107194. doi: 10.1016/j.compag.2022.107194

[B25] Wang J. Huang X. Yang R. Yuan J. Wang D. Chen J.. (2023). Study on usage reduction of glyphosate mixed with a novel protoporphyrinogen oxidase inhibitor-X18002. Chin. J. Pestic. Sci. 25 (04), 817–828. doi: 10.16801/j.issn.1008-7303.2023.0060

[B26] Wang A. Peng T. Cao H. Xu Y. Wei X. Cui B. (2022). TIA-YOLOv5: An improved YOLOv5 network for real-time detection of crop and weed in the field. Front. Plant Sci. 13. doi: 10.3389/fpls.2022.1091655 PMC9815699 36618638

[B27] Wu F. Yang Z. Mo X. Wu Z. Tang W. Duan J.. (2023). Detection and counting of banana bunches by integrating deep learning and classic image-processing algorithms. Comput. Electron. Agric. 209, 107827. doi: 10.1016/j.compag.2023.107827

[B28] Yang J. Wang Y. Chen Y. Yu L. (2022). Detection of weeds growing in Alfalfa using convolutional neural networks. Agron. 12 (6) 1459 doi: 10.3390/agronomy12061459

[B29] Yu Y. Wang B. Shi J. Li X. (2005). Design and experimental study of combined-charging hydraulic electrostatic spraying box. Trans. Chin. Soc Agric. Eng. 21 (12), 85–88.

[B30] Yue J. Yang G. Li C. Li Z. Wang Y. Feng H.. (2017). Estimation of Winter Wheat AboveGround Biomass Using Unmanned Aerial Vehicle- Based Snapshot Hyperspectral Sensor and Crop Height Improved Models. Remote Sens. 9 (7), 708. doi: 10.3390/rs9070708

[B31] Zhang Y. Li R. Mu X. Ren H. (2021). Extraction of paddy rice planting areas based on multi-temporal GF-6 remote sensing images. Trans. Chin. Soc Agric. Eng. 37 (17), 189–196. doi: 10.11975/j.issn.1002-6819.2021.17.021

[B32] Zhang H. Wang Z. Guo Y. Ma Y. Cao W. Chen D.. (2022). Weed detection in peanut fields based on machine vision. Agric. 12 (10). doi: 10.3390/agriculture12101541

[B33] Zhang B. Zhao D. (2023). An ensemble learning model for detecting soybean seedling emergence in UAV imagery. Sensors. 23 (15). doi: 10.3390/s23156662 PMC10422598 37571446

[B34] Zhu Z. He Y. Li W. Cai Z. Wang Q. Ma M. (2023). Improved YOLOv7 model for duck egg recognition and localization in complex environments. Trans. Chin. Soc Agric. Eng. 39 (11), 274–285. doi: 10.11975/j.issn.1002-6819.202303181

